# Clinical diagnosis of diabetes using machine learning and surface-enhanced Raman spectroscopy liquid biopsy: an exploratory study

**DOI:** 10.1039/d5na00905g

**Published:** 2025-10-28

**Authors:** Allah Ditta, Peiying Wu, Rui Zhang, Haq Nawaz, Muhammad Irfan Majeed, Sima Rezvantalab, Sara Mihandoost, Eva Miriam Buhl, Stephan Rütten, Fabian Kiessling, Twan Lammers, Roger M. Pallares

**Affiliations:** a Institute for Experimental Molecular Imaging, RWTH Aachen University Hospital Aachen 52074 Germany rmoltopallar@ukaachen.de; b Department of Chemistry, University of Agriculture Faisalabad Faisalabad 38000 Pakistan; c Chemical Engineering Department, Urmia University of Technology Urmia 57166-419 Iran; d Electrical Engineering Department, Urmia University of Technology Urmia 57166-419 Iran; e Electron Microscope Facility, Institute for Pathology, RWTH Aachen University Hospital Aachen 52074 Germany

## Abstract

The impact of diabetes on global health is increasing, underscoring the need for early and accurate diagnosis to prevent severe complications. Nevertheless, conventional diagnostic approaches, such as glycated hemoglobin testing and oral glucose tolerance tests, often lack sensitivity or specificity, particularly for detecting the disease at an early stage. In this exploratory clinical study, we present a promising alternative, label-free surface-enhanced Raman spectroscopy (SERS), which enables rapid, non-invasive biochemical analysis of liquid samples. Using gold nanoparticles as substrates, we applied label-free SERS to clinical serum samples for diabetes diagnosis. Because label-free SERS analysis of biological samples yields complex spectra, we developed a machine learning workflow tailored to clinical samples, exploring four different machine learning models in combination with synthetic data augmentation. This approach achieved classification accuracies of 96% and 94% for the healthy and diabetes groups, respectively. Our results demonstrate the benefits of integrating label-free SERS and machine learning models for efficient, accurate diabetes diagnosis *via* liquid biopsy, offering a powerful tool to enhance detection and potentially improve patient outcomes worldwide.

## Abstract 2

This study demonstrates the benefits of integrating label-free SERS and machine learning for efficient, accurate diabetes diagnosis *via* liquid biopsy.

## Graphical abstract

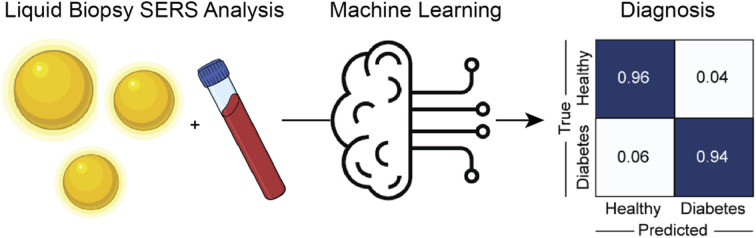

## 1 Introduction

Diabetes is a group of metabolic disorders marked by high blood sugar levels (hyperglycemia) due to insufficient insulin production or impaired insulin action.^1^ 537 million people aged 20 to 79 years old had diabetes in 2021 worldwide, and this number is expected to rise to 783 million by 2045.^2^ Chronic hyperglycemia is associated with long-term damage, dysfunction, and failure of several organs, including the eyes, kidneys, and heart.^3^ Diabetes is classified into type 1 and type 2. The former is caused by the autoimmune destruction of β-cells, which usually leads to complete insulin deficiency, while the latter results from a progressive decline in β-cell insulin secretion, often occurring alongside insulin resistance.^4,5^

Early and accurate diagnosis of diabetes is essential to prevent complications, such as cardiovascular disease, kidney failure, and retinopathy, which can lead to disability or premature death.^6^ However, the effectiveness of the most frequently used diagnostic assays, namely glycated hemoglobin (HbA1c) and fasting plasma glucose (FPG) tests, which measure blood glucose levels, is limited. For example, HbA1c and FPG tests can present sensitivities below 60% for diabetes diagnosis, depending on the patient cohort.^7^ Moreover, they are inadequate to detect diabetes at an early stage.^8^ Alternative protocols based on enzyme-linked immunosorbent assays and mass spectrometry, which detect other biomarkers, such as insulin, C-peptide, adiponectin, and inflammatory cytokines, have been explored for the diagnosis of diabetes; however, they are intensive in cost and time.^9–11^ Therefore, there is a real medical need for developing a novel approach to diagnose diabetes with high sensitivity and specificity, while being affordable, rapid, and straightforward.

Raman spectroscopy is a non-invasive analytical method that provides extensive information about the structure and composition of biomolecules.^12,13^ Raman spectroscopy is known for its ability to generate molecular fingerprints of analytes, and has been extensively used to investigate biological materials, such as the molecular composition of plasma samples.^14,15^ Nevertheless, Raman spectroscopy relies on the inelastic scattering of photons, which is very inefficient, yielding weak signals and low sensitivities.^16^ The signal intensity in Raman spectroscopy can be amplified by placing the analytes in the near fields of plasmonic materials.^17,18^ For example, gold nanoparticles (AuNPs) can enhance the Raman intensities of molecules located at their surface by more than 10^8^-fold.^19^ This approach is known as surface-enhanced Raman spectroscopy (SERS) and can achieve limits of detection in the zeptomole range and (even) single-molecule detection.^20,21^ Notably, water does not interfere with SERS measurements, which are quick, only requiring a few seconds to record a full spectrum. As a result, SERS has been widely exploited to analyze environmental, chemical, pharmaceutical, and medical samples.^22^ Most SERS approaches rely on the use of Raman tags and targeting agents to selectively detect specific analytes. Nevertheless, label-free protocols, where SERS is used to probe whole samples rather than characterizing a specific analyte, are gaining momentum, particularly in biomedicine. For example, label-free SERS has been employed to identify and discriminate protein biomarkers and disease-associated pathogens.^23–26^ Although label-free SERS protocols are quick and affordable, they result in complex Raman spectra, which are very hard to discern and interpret when the analyzed samples have complex compositions, such as liquid biopsy samples. Furthermore, most label-free SERS methods rely on highly refined gold substrates obtained through nanofabrication techniques, which improve measurement reliability but limit widespread use.^27^ While colloidal AuNPs do allow SERS measurements, they tend to yield spectra with smaller intensities, challenging the analysis and classification of complex samples based on spectroscopical features.^28,29^ Developing methods that can provide robust diagnostic information with AuNPs would be highly advantageous, since they can be easily synthesized *via* colloidal one-pot protocols, even in low-resource environments. Hence, analysis methods are necessary to identify spectral characteristics that can discriminate samples and obtain diagnostic information from label-free SERS biosensing with colloidal AuNPs.

Machine learning (ML) is becoming a fundamental tool in biosensing and diagnosing large datasets, identifying patterns and relationships between healthy and disease groups, and predicting patient conditions.^30^ For example, random forest algorithms have been used to analyze gene expression data, identifying gene signatures linked to various cancer types and highlighting sequence candidates found in circulating tumor DNA for liquid biopsy-based diagnosis.^31^ ML models have also been used to handle single-cell sequencing data^32^ and to detect patterns in immune cell populations and cytokine levels for more accurate classification of autoimmune conditions, improving patient outcomes through timely interventions.^33^ Moreover, ML has expanded the functionality of Raman spectroscopy, providing information otherwise inaccessible due to sample complexity, such as label-free single-cell analysis and incubation-free determination of tuberculosis drug resistance strains.^34,35^ Because the sample size defines the prediction quality of ML during the training phase, data augmentation strategies are often necessary to overcome the limitations of data scarcity.^36–38^ In the context of assessing diabetes using SERS liquid biopsy, we hypothesized that ML algorithms combined with data augmentation could identify spectral characteristics to obtain clinically relevant diagnostic information with AuNPs.

In this study, we demonstrate that integrating label-free SERS and ML models can be used to accurately diagnose diabetes with serum samples of patients. Augmentation with synthetic data improved the performance across the different models, reaching classification accuracies up to 96% and 94% for the healthy and diabetic groups, respectively. This work offers a new approach to rapidly diagnose diabetes, as well as potentially other metabolic diseases.

## 2 Experimental section

### 2.1 Synthesis of AuNPs

AuNPs were synthesized using the Turkevich method, a widely used procedure for producing colloidal gold through the chemical reduction of gold salts with trisodium citrate (99% Na_3_C_6_H_5_O_7_, Sigma-Aldrich, USA).^39–43^ First, hydrogen tetrachloroaurate (99% HAuCl_4_, Sigma-Aldrich, USA), the gold precursor, was dissolved in 20 mL of deionized water to create a 1 mM gold salt solution. Meanwhile, 12.5 mg of trisodium citrate, serving as a reducing and capping agent, was dissolved in 50 mL of deionized water. This solution was then heated in a round-bottom flask and continuously stirred with a magnetic stirrer.

Once the temperature reached 100 °C, 1 mL of the prepared gold salt solution was added to the citrate solution, changing the color to pale yellow. Subsequently, five 1 mL aliquots of the gold salt solution were added to the boiling trisodium citrate solution at 20 minute intervals while maintaining continuous stirring. By the end of this process, the solution turned dark red.

After this, heating and stirring were stopped, allowing the solution to cool to room temperature. The resulting solution, containing the synthesized AuNPs, was stored at 4 °C for future use.

### 2.2 Characterization of AuNPs

Transmission electron microscopy (TEM) was employed to characterize the size and morphology of the AuNPs. Initially, the AuNPs were centrifuged at 9000 rpm for 10 min and then resuspended in deionized water. The resuspended solution was drop-cast onto a 200-mesh carbon-coated copper grid (Plano GmbH, Germany). The grids were allowed to air dry overnight at room temperature before examination with a Hitachi TEM system operating at 100 kilovolts. The composition of the AuNPs was further confirmed using energy-dispersive spectroscopy (EDS). Additionally, the optical properties of the AuNPs were evaluated using an Infinite Pro microplate reader (Tecan, Switzerland).

### 2.3 Preparation of blood serum samples

52 blood serum samples were collected from Nishtar Medical University, Multan, Pakistan. This collection included 10 samples from healthy patients and 42 samples from individuals with confirmed diabetes. An anonymized description of the patients is provided in Table S1. Samples were collected from both female and male patients. None had comorbidities or were under medication, as blood was obtained at the time of initial clinical diagnosis. The Institutional Ethical Review Board at the Nishtar Medical University approved the sample collection and use for developing new sensing technologies. Informed consent was obtained from all subjects. The clinical study was registered in clinicaltrials.gov (http://clinicaltrials.gov) (NCT06862778). The study focused on serum, a component of blood obtained through centrifugation after removing cells and clotting factors. The serum is free of cellular elements and primarily consists of proteins and other biologically active compounds, making it a more suitable sample for targeted analysis.^44^ The serum samples were further treated with 100 kDa filtering devices (Amicon ultra centrifugal filters, Sigma-Aldrich, USA), for 30 minutes at 6500 rpm.

### 2.4 SERS measurements

Each serum sample (20 μL) was combined with an equal volume of AuNPs in an Eppendorf tube. The resulting mixtures were ultrasonicated at 28 kHz and 150 W for 30 minutes to ensure homogeneous mixing between the AuNPs and the serum samples. After mixing, the samples were incubated for two hours at 4 °C.

Next, 20 μL of the prepared samples were placed onto an aluminum slide for measurement following a previously established protocol.^45,46^ The spectra were recorded using an Optosky Raman Microscope Spectrometer (model ATR8300BS), which was equipped with a 785 nm diode laser as the Raman excitation source. The excitation light was focused onto the sample using a 20× objective lens, with a laser power set to 250 mW to optimize the signal-to-noise ratio. A 30 second integration period was used for each spectrum. Fifteen spectra were collected for each sample at room temperature, with the Raman shift range set between 300 cm^−1^ and 1600 cm^−1^ to capture relevant molecular vibrational information.

Fifteen spectra per sample were recorded to obtain the mean spectral plot for each sample. This approach reduces noise and improves the signal-to-noise ratio, better representing the characteristic vibrational bands in the samples.

### 2.5 Data pre-processing

The raw data from the SERS experiments were processed using MATLAB R2023a (The MathWorks, USA) and standard chemometric techniques that utilized custom-developed algorithms. The pre-processing steps involved removing the aluminum substrate signal, performing baseline correction, normalizing the data vectors, and applying smoothing through Savitzky–Golay filtering (Fig. S1). The filtering parameters were set to a 17th-order polynomial with a 14-point window width.

### 2.6 Multivariate data analysis

The changes in the SERS spectral features of the samples were analyzed using multivariate data analysis techniques, specifically principal component analysis (PCA) and mean spectral plots. PCA is a statistical method that simplifies multivariate data analysis by reducing a large number of correlated variables into a smaller set of uncorrelated variables. This technique helps identify patterns and relationships within the dataset. The dimensionality of the SERS data was reduced to highlight key principal components that distinguish between healthy samples and those with diabetes, while preserving the variability of the data.^47^

### 2.7 Machine learning

We employed four ML models for SERS spectral classification: K-nearest neighbors (KNN), artificial neural networks (ANN), support vector machines (SVM), and quadratic discriminant analysis (QDA), each chosen for its unique strengths. KNN (with a *K* value of 5) was selected for its simplicity and effectiveness with small to moderate datasets, adapting well to diverse data distributions.^48^ ANN excelled at modeling complex, non-linear relationships, utilizing architectures with rectified linear unit (ReLU) and sigmoid functions, and benefiting from careful tuning for efficiency.^49^ SVM is effective in high-dimensional spaces and adaptable through linear or RBF kernels.^50^ We optimized SVM parameters such as regularization (C) and kernel coefficient (gamma) to enhance the model accuracy. QDA is effective in modeling distinct covariance structures for each class.^51^ For QDA, we used regularization to ensure stable results in varying class distributions. Notably, these classification models were also selected because they show outstanding performance with small datasets.^52,53^ ML analysis was conducted by using the entire SERS spectra as input features for model training and evaluation. Each sample consisted of 15 spectra with 1499 features, corresponding to the number of vibrational modes observed in each spectrum. No dimensionality reduction, including PCA, was performed before training. This approach maintained full spectral information for classification using ML models. The synthetic minority over-sampling technique (SMOTE) is an oversampling method designed to address class imbalance in datasets.^54^ SMOTE generates synthetic samples for the minority class to balance the dataset, rather than just duplicating instances. It identifies minority instances and their k-nearest neighbors, creating new samples through interpolation between them.^55^ To ensure a good balance between healthy and diabetes samples, 480 healthy data points were generated with SMOTE to match the number of data points between both groups.

## 3 Results and discussion

**Fig. 1:**
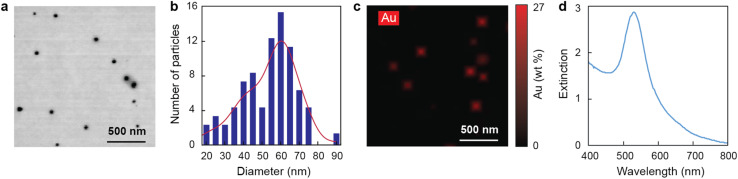
Characterization of AuNPs. (a) Transmission electron microscopy micrographs, (b) size distribution, and (c) micrographs with energy-dispersive X-ray spectroscopy signal of gold (Au weight (wt)%) highlighted in red of AuNPs. (d) Extinction spectra of AuNPs in solution.

AuNPs were synthesized using the Turkevich method, which involves the chemical reduction of gold salts with citrate. The resulting AuNPs were spherical and had an average diameter of 56 ± 5 nm (Fig. 1a and b). Energy-dispersive X-ray spectroscopy mapping confirmed that the particles were composed of gold (Fig. 1c). Additionally, the AuNPs exhibited an extinction band centered around 529 nm (Fig. 1d), which is consistent with the reported position of the localized surface plasmon resonance of spherical AuNPs.^56,57^

**Fig. 2:**
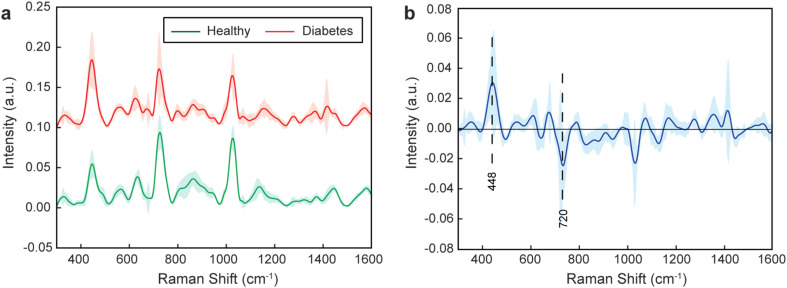
SERS characterization of healthy and diabetic patients. (a) Mean Raman spectra of all samples within a group as determined by SERS. (b) Difference in mean spectrum between the two groups (healthy – diabetes) with main differential peaks highlighted. The sharp lines represent the mean spectra and the pale areas represent one standard deviation of the measurements.

Next, we employed the AuNPs to perform the SERS analysis of the healthy and diabetic liquid biopsy samples. A total of 52 blood serum samples, consisting of 10 samples from healthy volunteers and 42 samples from diabetic patients, were obtained from the Nishtar Medical University Multan (Pakistan). Before use, the samples were filtered with 100 kDa filtering devices to isolate low molecular weight biomolecules, as most of the potential biomarkers responsible for diabetes are under 100 kDa.^58^ The AuNPs and filtered serum samples were mixed continuously for 30 minutes at 4 °C, before being deposited on aluminum substrates, and their Raman spectra recorded. 15 spectra were recorded for each sample to obtain a better representation of their characteristic vibrational bands. To identify spectral differences between healthy and diabetic patient samples, we determined the mean spectra of all the samples within a group (Fig. 2a). The difference in mean spectrum between healthy and diabetic patient samples revealed significant variations across multiple peaks (Fig. 2b), which tend to be associated with biomolecular composition alterations. However, because the serum is a complex matrix with many different components displaying overlapping peaks, assigning each peak to a biomolecule or a group is challenging.

**Fig. 3:**
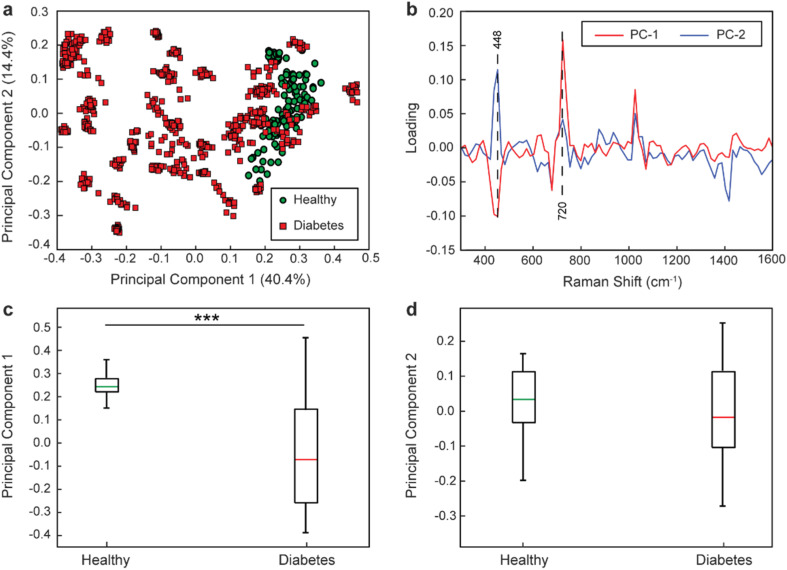
Spectral differences between healthy and diabetic patient samples based on SERS measurements. (a) PCA of healthy and diabetic patient samples. The principal component 1 and 2 describe 40.4% and 14.4% of the total variance, respectively. The plot presents 52 samples with 15 data points (spectra) per sample. (b) Loadings of the first and second principal components (PC-1 and PC-2, respectively). Average PCA scores of (c) the first and (d) the second principal components. The colored bars and black squares represent the means and the interquartile ranges of the data. *** indicate groups with large effect sizes (Cohen's *d* > 1.4, two-tailed *t*-test).

To further differentiate the two groups, we carried out PCA, which reduced the dimensionality of the high-dimensional datasets by transforming the original variables into a smaller set of uncorrelated variables known as principal components.^59^Fig. 3a presents the PCA plot of all measured spectra, and shows a fair separation between groups. The horizontal and vertical axes correspond to the first (PC-1) and second (PC-2) principal components, which explained 40.4% and 14.4% of the total variance, respectively. Hence, the first principal component accounted for the largest variance, representing the most important patterns in the SERS spectra. All healthy samples were located on the positive side of the horizontal axis (PC-1), with values above 0.15, whereas most diabetic patient samples (75%) had values smaller than that. 25% of the diabetic patient data points, however, partially overlapped with the healthy data region on the PC-1 axis, likely due to serum variability factors, such as diet, blood glucose levels, and degree of diabetes. To better understand the differences between the two groups, we analyzed the PCA loading plots (Fig. 3b), which showed clear differences, particularly along PC-1. For instance, strong variations were observed in the 448 and 720 cm^−1^ peaks, which tend to be associated with cholesterol and nucleic acid.^60,61^ The PCA score analysis for the first two components (Fig. 3c and d) showed that despite the partial overlap between the healthy and diabetes groups, they were statistically different in PC-1 with large effect sizes (Cohen's *d* > 1.4 and *p* < 0.001). Those differences are enough to overall distinguish both groups based on PCA coordinates, however, they are likely to yield limited sensitivity and specificity when using PCA for the diagnosis of new samples.

Next, we explored whether ML could improve the diagnostic capabilities of our SERS approach. Four different models commonly used in the analysis of sensing data were explored, namely KNN, ANN, QDA, and SVM. For each model evaluation, 80% of the data from the healthy and diabetic patient groups were randomly selected for training, with the remaining 20% being reserved for testing. The dataset was split into training and testing sets before any pre-processing, such as normalization. This approach prevents data leakage by ensuring the test set does not influence training, preserving the integrity of the evaluation and providing an unbiased assessment of the performance of the models. The normalization parameters, such as mean and standard deviation, were calculated using only the training data, and then applied to both the training and test sets. The performances of the models were evaluated with 5-fold cross-validation, averaging the results to provide a robust estimate of model performance. Furthermore, since ML model performance strongly depends on data size, and our sample pool was imbalanced with a greater number of diabetic patient samples compared to healthy ones (42 *vs.* 10), we also explored a data generation method, named SMOTE. This technique helps to reduce the bias that models may develop toward the majority class when faced with imbalanced data.^62^ The data augmentation with SOMTE was applied exclusively to the training data within each fold of the cross-validation procedure to prevent data leakage. Hence, the test sets (untouched real data) were kept completely independent and unaffected by the SMOTE process, ensuring that the performance assessment of the models reflect their true generalization capabilities without any information leakage. Hence, 480 synthetic healthy data points were generated using SMOTE to balance the two groups. As shown by PCA (Fig. S2), the synthetic data broadly occupied the same regions of feature space as the original healthy data but did not perfectly overlap, suggesting that the generated data captured the underlying distribution without simply memorizing individual records.

**Fig. 4:**
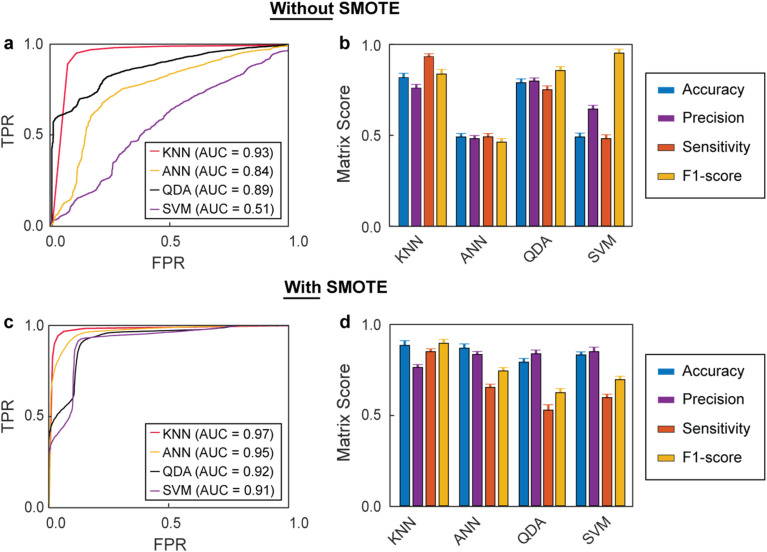
Receiver operating characteristic (ROC) curves of the different models, and their matrix scores (a) ROC curves and area under the curve (AUC) values for all models without data generation with SMOTE. The curves display true positive rates (TPR) against the false positive rates (FPR). (b) Matrix scores for all ML models without data generation with SMOTE. (c) ROC curves and their AUC values for all ML models with data generation with SMOTE. (d) Matrix scores for all ML models with data generation with SMOTE. Error bars represent one standard deviation across cross-validation folds. For each model, 80% of the dataset (8 healthy and 34 diabetic patients) was used for training, and 20% (2 healthy and 8 diabetic patients) was reserved for testing, from a total of 52 samples.

In the absence of synthetic data, the KNN model achieved an area under the curve (AUC) of 0.93 in the receiver operating characteristic (ROC) curves (Fig. 4a), the highest value among the different models, which indicated robust classification performance. The ANN, QDA, and SVM achieved poorer performances with AUC values of 0.84, 0.89, and 0.51, respectively. These results highlighted the large variability in performance across models, with SVM particularly struggling with the (imbalanced) data sets. Fig. 4b further breaks down the performance metrics, including accuracy, precision, sensitivity, and F1-score. KNN performed well in all four categories, with values ranging between 0.76 and 0.93. Interestingly, although ANN presented relatively good AUC values, it displayed the lowest performance metrics, with values ranging between 0.48 and 0.50. A high AUC and poor matrix scores, as observed for the ANN model, can indicate class imbalance. This situation arises when the model performs well overall but struggles with the minority class, such as the healthy samples.^63^ QDA, on the other hand, presented relatively good performance metrics (between 0.75 and 0.85), consistent with its good AUC. Lastly, SVM presented poor metric performances except for F1-score, which was very high (0.94). Next, we explored the impact of including synthetic data on the performance of the models. Notably, all models' performances improved with the generated data, achieving AUC values above 0.90, and KNN was again the best-performing model with an AUC value of 0.97 (Fig. 4c). Furthermore, SMOTE consistently narrowed the 95% confidence intervals across models (Table S2), indicating enhanced stability and generalizability. KNN was also the model with the best performance metrics, as shown in Fig. 4d. Although including synthetic data with the SMOTE method improved all metrics, it had the strongest effects on accuracy and precision, with values above 0.80 for all models. Overall, combining data generation with SMOTE and the KNN model achieved the highest AUC and demonstrated superior values across performance metrics, making it the best choice for enhancing diagnostic accuracy in imbalanced datasets. Furthermore, these results also highlighted the importance of addressing class imbalance to improve the reliability and effectiveness of the models.

To better assess the impact of data generation on the model performances, particularly in terms of generalization, we compared the AUC scores between the training and test sets (AUC mean differences). After 50 iterations, in absence of synthetic data, all models showed mean differences below 0.1, suggesting no significant overfitting (Fig. S3). The values decreased as synthetic data was introduced for training, indicating better generalization. The KNN model with SMOTE-generated data was the best combination, with an AUC mean difference value of 0.018, indicating excellent generalization (Fig. S4). Notably, for SVM, although the AUC rose sharply from 0.51 to 0.91 with data generation, the mean training–testing AUC difference remained nearly unchanged (0.096 *vs.* 0.093). This reflects that the added data improved both training and testing performance to a similar extent, yielding a substantial gain in absolute accuracy but little change in the relative generalization gap.

**Fig. 5:**
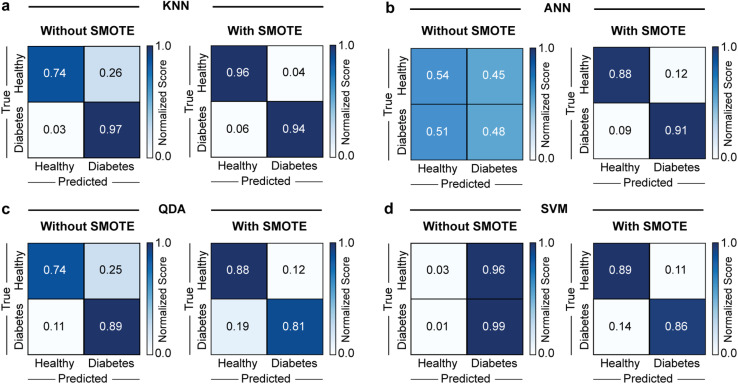
Confusion matrices from 5-fold cross-validation for all different models without and with synthetic data. Normalized scores for the different models, (a) KNN, (b) ANN, (c) QDA, and (d) SVM models, without and with data generated with SMOTE. Because the confusion matrices are row-normalized, the values along the diagonal correspond directly to the recall for reach class.

Finally, Fig. 5 displays the confusion matrices for the four models without and with synthetic data under 5-fold cross-validation. Consistent with the previous analyses, including synthetic data improved the overall performance of all models. The best-performing model without and with data generation was KNN. Its classification accuracy for healthy and diabetic patient samples was 74% and 97% without data generation. The inclusion of generated data with SMOTE improved the accuracy in the classification of healthy samples to 96% and slightly decreased the accuracy for diabetic patient samples to 94%, which resulted in better overall diagnostic performance. These accuracy results outperformed those of gold standard methods, such as HbA1c and fasting plasma glucose tests, which typically yield sensitivities of up to 80% and AUC values ranging from 0.80 to 0.92.^7,64^ Although the results with the other models followed similar trends, their accuracies were consistently lower than that of KNN. Interestingly, for ANN, the ROC analysis indicated relatively strong overall discriminative ability (AUC of 0.84, Fig. 4a) without data generation. In contrast, the confusion matrix showed poor class-wise accuracies (TNR of 0.54 and TPR of 0.48, Fig. 5b). This apparent discrepancy reflects the threshold-independent nature of AUC *versus* the threshold dependence of confusion matrices, suggesting that although the model could separate classes effectively across thresholds, the applied cut-off was suboptimal and limited its classification performance. Nevertheless, this study was constrained by the limited number of patient samples, which may restrain the robustness of the predictive model. Therefore, the findings should be considered exploratory, and future studies with larger patient cohorts will be necessary to assess the generalizability of this approach.

Taken together these results demonstrated that SERS and ML could be used to diagnose diabetic samples with high accuracy (above 94%). Among the different models, KNN consistently performed the best. Furthermore, including synthetic data generated with the SMOTE method improved the performance of all models, as it addressed the class imbalance and particularly improved classification accuracy for the minority class (healthy) samples.

## 4 Conclusions

In summary, this exploratory clinical study demonstrates the integration of label-free SERS with ML models for the diagnosis of diabetes *via* liquid biopsy analysis. Four ML models were evaluated, namely KNN, ANN, QDA, and SVM, with KNN consistently outperforming the others across most performance metrics. To enhance classification performance, synthetic data were generated using the SMOTE method, resulting in improved model accuracy. Notably, KNN with SMOTE-augmented data achieved classification accuracies of up to 96% for healthy samples and 94% for diabetes samples. These findings indicate that the combination of label-free SERS and ML, particularly when augmented with synthetic data, holds promise for the rapid and non-invasive diagnosis of diabetes and potentially other metabolic diseases.

## Author contributions

AD (conceptualization, investigation, formal analysis, writing – original draft); PW (formal analysis), RZ (investigation); HN (methodology); MIM (methodology); SR (methodology); SM (methodology); EMB (formal analysis); SR (formal analysis); FK (supervision); TL (supervision); RMP (conceptualization, supervision, writing – review & editing). All the authors read and approved the submitted version of the manuscript.

## Conflicts of interest

The authors have no relevant affiliations or financial involvement with any organization or entity with a financial interest in or financial conflict with the subject matter or materials discussed in the manuscript.

## Supplementary Material

NA-OLF-D5NA00905G-s001

## Data Availability

The data supporting this article have been included as part of the SI. Supplementary information is available. See DOI: https://doi.org/10.1039/d5na00905g.

## References

[cit1] Ta S. (2014). Diagnosis and classification of diabetes mellitus. Diabetes care.

[cit2] Ogurtsova K., Guariguata L., Barengo N. C., Ruiz P. L.-D., Sacre J. W., Karuranga S., Sun H., Boyko E. J., Magliano D. J. (2022). IDF diabetes Atlas: Global estimates of undiagnosed diabetes in adults for 2021. Diabetes Res. Clin. Pract..

[cit3] Association A. D. (2014). Diagnosis and classification of diabetes mellitus. Diabetes care.

[cit4] Association A. D. (2018). 2. Classification and diagnosis of diabetes: standards of medical care in diabetes—2018. Diabetes care.

[cit5] Yoon J.-W., Jun H.-S. (2005). Autoimmune destruction of pancreatic β cells. Am. J. Therapeut..

[cit6] James M. T., Hemmelgarn B. R., Tonelli M. (2010). Early recognition and prevention of chronic kidney disease. Lancet.

[cit7] Kaur G., Lakshmi P., Rastogi A., Bhansali A., Jain S., Teerawattananon Y., Bano H., Prinja S. (2020). Diagnostic accuracy of tests for type 2 diabetes and prediabetes: A systematic review and meta-analysis. PLoS One.

[cit8] Ortiz-Martínez M., González-González M., Martagón A. J., Hlavinka V., Willson R. C., Rito-Palomares M. (2022). Recent developments in biomarkers for diagnosis and screening of type 2 diabetes mellitus. Curr. Diabetes Rep..

[cit9] Chen X., Stein T. P., Steer R. A., Scholl T. O. (2019). Individual free fatty acids have unique associations with inflammatory biomarkers, insulin resistance and insulin secretion in healthy and gestational diabetic pregnant women. BMJ Open Diabetes Res. Care.

[cit10] Eldjarn G. H., Ferkingstad E., Lund S. H., Helgason H., Magnusson O. T., Gunnarsdottir K., Olafsdottir T. A., Halldorsson B. V., Olason P. I., Zink F. (2023). Large-scale plasma proteomics comparisons through genetics and disease associations. Nature.

[cit11] Lin D., Alborn W. E., Slebos R. J., Liebler D. C. (2013). Comparison of protein immunoprecipitation-multiple reaction monitoring with ELISA for assay of biomarker candidates in plasma. J. Proteome Res..

[cit12] Kanwal N., Rashid N., Majeed M. I., Nawaz H., Amber A., Zohaib M., Bano A., Albekairi N. A., Alshammari A., Shahzadi A. (2024). Surface-enhanced Raman spectroscopy for the characterization of xylanases enzyme. Spectrochim. Acta, Part A.

[cit13] Krynicka P., Koulaouzidis G., Skonieczna-Żydecka K., Marlicz W., Koulaouzidis A. (2025). Application of Raman Spectroscopy in Non-Invasive Analysis of the Gut Microbiota and Its Impact on Gastrointestinal Health. Diagnostics.

[cit14] Birech Z., Mwangi P. W., Bukachi F., Mandela K. M. (2017). Application of Raman spectroscopy in type 2 diabetes screening in blood using leucine and isoleucine amino-acids as biomarkers and in comparative anti-diabetic drugs efficacy studies. PLoS One.

[cit15] Zhang S., Qi Y., Tan S. P. H., Bi R., Olivo M. (2023). Molecular fingerprint detection using Raman and infrared spectroscopy technologies for cancer detection: a progress review. Biosensors.

[cit16] Kneipp K., Kneipp H., Itzkan I., Dasari R. R., Feld M. S. (2002). Surface-enhanced Raman scattering and biophysics. J. Phys.: Condens. Matter.

[cit17] Alvarez-Puebla R. A., Liz-Marzán L. M. (2010). SERS-based diagnosis and biodetection. Small.

[cit18] Pallares R. M., Thanh N. T. K., Su X. (2019). Sensing of Circulating Cancer Biomarkers with Metal Nanoparticles. Nanoscale.

[cit19] Saleem M., Nawaz H., Majeed M. I., Rashid N., Anjum F., Tahir M., Shahzad R., Sehar A., Sabir A., Rafiq N. (2023). Surface-enhanced Raman spectroscopy (SERS) for the characterization of supernatants of bacterial cultures of bacterial strains causing sinusitis. Photodiagnosis Photodyn. Ther..

[cit20] Qiu Y., Kuang C., Liu X., Tang L. (2022). Single-molecule surface-enhanced Raman spectroscopy. Sensors.

[cit21] Rodríguez-Lorenzo L., Álvarez-Puebla R. A., Pastoriza-Santos I., Mazzucco S., Stéphan O., Kociak M., Liz-Marzán L. M., García de Abajo F. J. (2009). Zeptomol detection through controlled ultrasensitive surface-enhanced Raman scattering. J. Am. Chem. Soc..

[cit22] Peng J., Song Y., Lin Y., Huang Z. (2024). Introduction and Development of Surface-Enhanced Raman Scattering (SERS) Substrates: A Review. Nanomaterials.

[cit23] Kong K. V., Leong W. K., Lam Z., Gong T., Goh D., Lau W. K. O., Olivo M. (2014). A Rapid and Label-free SERS Detection Method for Biomarkers in Clinical Biofluids. Small.

[cit24] Arabi M., Ostovan A., Zhang Z., Wang Y., Mei R., Fu L., Wang X., Ma J., Chen L. (2021). Label-free SERS detection of Raman-Inactive protein biomarkers by Raman reporter indicator: Toward ultrasensitivity and universality. Biosens. Bioelectron..

[cit25] Ditta A., Zhang R., Nawaz H., Majeed M. I., He S., Zhuang Z., Rütten S., Shahzadi A., Yaseen S., Kiessling F., Hu J., Lammers T., Pallares R. M. (2025). An exploratory clinical study of the diagnosis and staging of typhoid fever using label-free surface-enhanced Raman spectroscopy liquid biopsy. Spectrochim. Acta, Part A.

[cit26] Tariq A., Javed M. R., Majeed M. I., Nawaz H., Rashid N., Yousaf S., Ijaz A., Huda N. u., Tahseen H., Naman A., Aziz S., Tariq R., Pallares R. M. (2024). Characterization of Aspergillus niger DNA by Surface-Enhanced Raman Spectroscopy (SERS) with Principal Component Analysis (PCA) and Partial Least Square Discriminant Analysis (PLS-DA) with Application for the Production of Cellulase. Anal. Lett..

[cit27] Mosier-Boss P. A. (2017). Review of SERS substrates for chemical sensing. Nanomaterials.

[cit28] Tantra R., Brown R. J., Milton M. J. (2007). Strategy to improve the reproducibility of colloidal SERS. J. Raman Spectrosc..

[cit29] Sloan-Dennison S., Wallace G. Q., Hassanain W. A., Laing S., Faulds K., Graham D. (2024). Advancing SERS as a quantitative technique: challenges, considerations, and correlative approaches to aid validation. Nano Convergence.

[cit30] Banerjee A., Maity S., Mastrangelo C. H. (2021). Nanostructures for biosensing, with a brief overview on cancer detection, IoT, and the role of machine learning in smart biosensors. Sensors.

[cit31] Flynn C. D., Chang D. (2024). Artificial intelligence in point-of-care biosensing: challenges and opportunities. Diagnostics.

[cit32] Hu D., Dong Z., Liang K., Yu H., Wang S., Liu X. (2024). High-order Topology for Deep Single-Cell Multiview Fuzzy Clustering. IEEE Trans. Fuzzy Syst..

[cit33] Kruta J., Carapito R., Trendelenburg M., Martin T., Rizzi M., Voll R. E., Cavalli A., Natali E., Meier P., Stawiski M. (2024). Machine learning for precision diagnostics of autoimmunity. Sci. Rep..

[cit34] Zhang Y., Chang K., Ogunlade B., Herndon L., Tadesse L. F., Kirane A. R., Dionne J. A. (2024). From genotype to phenotype: Raman spectroscopy and machine learning for label-free single-cell analysis. ACS Nano.

[cit35] Ogunlade B. , Tadesse L. F., Li H., Vu N., Banaei N., Barczak A. K., Saleh A. A., Prakash M. and Dionne J. A., Rapid, Antibiotic Incubation-free Determination of Tuberculosis Drug Resistance Using Machine Learning and Raman Spectroscopy, Proceedings of the National Academy of Sciences, 2024, 121, e2315670121 10.1073/pnas.2315670121 PMC11194509 38861604

[cit36] Wallace D., Delaney E., Keane M. T., Greene D. (2021). Artificial Intelligence and Complex Systems.

[cit37] Iglesias G., Talavera E., González-Prieto Á., Mozo A., Gómez-Canaval S. (2023). Data augmentation techniques in time series domain: a survey and taxonomy. Neural Comput..

[cit38] Wu P., Zhang R., Porte C., Kiessling F., Lammers T., Rezvantalab S., Mihandoost S., Pallares R. M. (2025). Machine learning to predict gold nanostar optical properties. Nanoscale Adv.

[cit39] Kimling J., Maier M., Okenve B., Kotaidis V., Ballot H., Plech A. (2006). Turkevich method for gold nanoparticle synthesis revisited. J. Phys. Chem. B.

[cit40] Oliveira A. E. F., Pereira A. C., Resende M. A. C., Ferreira L. F. (2023). Analytica.

[cit41] Wuithschick M., Birnbaum A., Witte S., Sztucki M., Vainio U., Pinna N., Rademann K., Emmerling F., Kraehnert R., Polte J. r. (2015). Turkevich in New Robes: Key Questions Answered for the Most Common Gold Nanoparticle Synthesis. ACS Nano.

[cit42] Zhang R., Thoröe-Boveleth S., Chigrin D. N., Kiessling F., Lammers T., Pallares R. M. (2024). Nanoscale engineering of gold nanostars for enhanced photoacoustic imaging. J. Nanobiotechnol..

[cit43] Pallares R. M., Choo P., Cole L. E., Mirkin C. A., Lee A., Odom T. W. (2019). Manipulating Immune Activation of Macrophages by Tuning the Oligonucleotide Composition of Gold Nanoparticles. Bioconjugate Chem..

[cit44] Tuck M. K., Chan D. W., Chia D., Godwin A. K., Grizzle W. E., Krueger K. E., Rom W., Sanda M., Sorbara L., Stass S. (2009). Standard operating procedures for serum and plasma collection: early detection research network consensus statement standard operating procedure integration working group. J. Proteome Res..

[cit45] Umar Hussain M., Kainat K., Nawaz H., Irfan Majeed M., Akhtar N., Alshammari A., Albekairi N. A., Fatima R., Amber A., Bano A., Shabbir I., Tahira M., Pallares R. M. (2024). SERS characterization of biochemical changes associated with biodesulfurization of dibenzothiophene using Gordonia sp. HS126-4N. Spectrochim. Acta, Part A.

[cit46] Anwer A., Shahzadi A., Nawaz H., Majeed M. I., Alshammari A., Albekairi N. A., Hussain M. U., Amin I., Bano A., Ashraf A., Rehman N., Pallares R. M., Akhtar N. (2024). Differentiation of different dibenzothiophene (DBT) desulfurizing bacteria via surface-enhanced Raman spectroscopy (SERS). RSC Adv..

[cit47] Wu X., Huang Y.-W., Park B., Tripp R. A., Zhao Y. (2015). Differentiation and classification of bacteria using vancomycin functionalized silver nanorods array based surface-enhanced Raman spectroscopy and chemometric analysis. Talanta.

[cit48] Hastie T. , Tibshirani R. and Friedman J., Prototype Methods and Nearest-Neighbors, The Elements of Statistical Learning, Springer, New York, USA, 2nd edn, 2009, pp. 459–483.

[cit49] Baxt W. G. (1995). Application of artificial neural networks to clinical medicine. Lancet.

[cit50] Christmann A. and Steinwart I., Support Vector Machines for Classification, Support Vector Machine, Springer, New York, USA, 1st edn, 2008, pp. 285–329.

[cit51] Jiang B., Wang X., Leng C. (2018). A direct approach for sparse quadratic discriminant analysis. J. Mach. Learn. Res..

[cit52] Erzina M., Trelin A., Guselnikova O., Dvorankova B., Strnadova K., Perminova A., Ulbrich P., Mares D., Jerabek V., Elashnikov R. (2020). Precise cancer detection via the combination of functionalized SERS surfaces and convolutional neural network with independent inputs. Sens. Actuators, B.

[cit53] Astantri P. F., Prakoso W. S. A., Triyana K., Untari T., Airin C. M., Astuti P. (2020). Lab-made electronic nose for fast detection of Listeria monocytogenes and Bacillus cereus. Vet. Sci..

[cit54] Ding Y., Sun Y., Liu C., Jiang Q. Y., Chen F., Cao Y. (2023). SeRS-Based Biosensors Combined with Machine Learning for Medical Application. ChemistryOpen.

[cit55] Adi Pratama F. R., Oktora S. I. (2023). Synthetic Minority Over-sampling Technique (SMOTE) for handling imbalanced data in poverty classification. Stat. J. IAOS.

[cit56] Bastús N. G., Comenge J., Puntes V. (2011). Kinetically controlled seeded growth synthesis of citrate-stabilized gold nanoparticles of up to 200 nm: size focusing versus Ostwald ripening. Langmuir.

[cit57] Shafabakhsh R., Zhang R., Thoröe-Boveleth S., Moosavifar M., Abergel R. J., Kiessling F., Lammers T., Pallares R. M. (2025). Gold Nanoparticle-Enabled Fluorescence Sensing of Gadolinium-Based Contrast Agents in Urine. ACS Appl. Nano Mater..

[cit58] Atta M. M., Kashif M., Majeed M. I., Nawaz H., Alshammari A., Albekairi N. A., Parveen A., Usman M., Salfi A. B., Lateef A. (2025). Surface-Enhanced Raman Spectroscopy for the Characterization of Blood Serum Samples of Chronic Kidney Disease by Using 100 kDa. Plasmonics.

[cit59] Li W., You Z., Cao D., Liu N. (2024). A machine learning-driven SERS platform for precise detection and analysis of vascular calcification. Anal. Methods.

[cit60] Lu Y., Lei B., Zhao Q., Yang X., Wei Y., Xiao T., Zhu S., Ouyang Y., Zhang H., Cai W. (2023). Solid-state Au nanocone arrays substrate for reliable SERS profiling of serum for disease diagnosis. ACS Omega.

[cit61] Shoukat Z., Atta R., Majeed M. I., Nawaz H., Rashid N., Alshammari A., Albekairi N. A., Shahzadi A., Yaseen S., Tahir A. (2025). SERS profiling of blood serum filtrate components from patients with type II diabetes using 100 kDa filtration devices. RSC Adv..

[cit62] Kivrak M., Avci U., Uzun H., Ardic C. (2024). The Impact of the SMOTE Method on Machine Learning and Ensemble Learning Performance Results in Addressing Class Imbalance in Data Used for Predicting Total Testosterone Deficiency in Type 2 Diabetes Patients. Diagnostics.

[cit63] Bartosch-Härlid A., Andersson B., Aho U., Nilsson J., Andersson R. (2008). Artificial neural networks in pancreatic disease. Br. J. Surg..

[cit64] Duong K. N. C., Tan C. J., Rattanasiri S., Thakkinstian A., Anothaisintawee T., Chaiyakunapruk N. (2023). Comparison of diagnostic accuracy for diabetes diagnosis: A systematic review and network meta-analysis. Front. Clin. Med..

