# Supplementary material for: Clinical diagnosis of diabetes using machine learning and surface-enhanced Raman spectroscopy liquid biopsy: an exploratory study
Source: Nanoscale Adv. 2025 Oct 28;7(23):7504–13. doi: 10.1039/d5na00905g (PMC12560141; doi:10.1039/d5na00905g)
Supplement: NA-007-D5NA00905G-s001 [file NA-007-D5NA00905G-s001.pdf]

Supplementary Information (SI) for Nanoscale Advances.
This journal is © The Royal Society of Chemistry 2025

- SUPPORTING INFORMATION -

Clinical Diagnosis of Diabetes Using Machine Learning and Surface-enhanced Raman Spectroscopy Liquid Biopsy: An Exploratory Study

Allah Ditta,[1] Peiying Wu,[1] Rui Zhang,[1] Haq Nawaz,[2] Muhammad Irfan Majeed,[2] Sima Rezvantalab,[3] Sara Mihandoost,[4] Eva Miriam Buhl,[5] Stephan Rütten,[5] Fabian Kiessling,[1] Twan Lammers,[1] and Roger M. Pallares[1,*]

[1] Institute for Experimental Molecular Imaging, RWTH Aachen University Hospital, Aachen 52074, Germany

[2] Department of Chemistry, University of Agriculture Faisalabad, Faisalabad (38000), Pakistan

[3] Chemical Engineering Department, Urmia University of Technology, Urmia 57166-419, Iran

[4] Electrical Engineering Department, Urmia University of Technology, Urmia 57166-419, Iran

[5] Electron Microscope Facility, Institute for Pathology, RWTH Aachen University Hospital, Aachen 52074, Germany

[*]Corresponding author: rmoltopallar@ukaachen.de

Table of Contents

Figure S1. Representative SERS spectra before (raw) and after pre-processing..2

Table S1. List of samples with gender, age, medication, and other disease...3

Figure S2. PCA analysis of original and synthetic data points from the healthy group.............................5

Table S2. 95% confidence intervals of model performance without and with SMOTE. 6

Figure S3. AUC mean difference plots without SMOTE-generated data for all models. 7

Figure S4. AUC mean difference plots with SMOTE-generated data for all models. 8

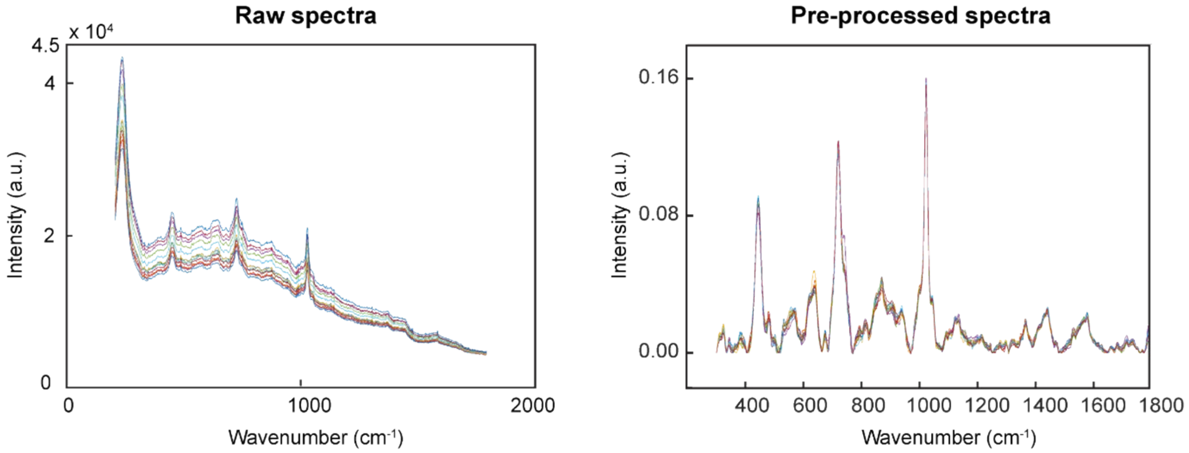

Figure S1. Representative SERS spectra before (raw) and after pre-processing. Pre-processing steps include normalization, baseline correction, and smoothing.

Table S1. List of samples with gender, age, medication, and other disease.					
Serial Number	Gender	Age	Diabetes	Medication	Other Disease
1	Female	41	Yes	No	No
2	Female	45	Yes	No	No
3	Female	45	Yes	No	No
4	Male	44	Yes	No	No
5	Male	40	Yes	No	No
6	Male	40	Yes	No	No
7	Male	40	Yes	No	No
8	Female	43	Yes	No	No
9	Male	40	Yes	No	No
10	Male	40	Yes	No	No
11	Male	43	Yes	No	No
12	Female	42	Yes	No	No
13	Female	40	Yes	No	No
14	Male	41	Yes	No	No
15	Male	40	Yes	No	No
16	Male	44	Yes	No	No
17	Male	41	Yes	No	No
18	Female	45	Yes	No	No
19	Female	40	Yes	No	No
20	Female	41	Yes	No	No
21	Male	45	Yes	No	No
22	Female	42	Yes	No	No
23	Female	42	Yes	No	No
24	Female	43	Yes	No	No
25	Female	41	Yes	No	No
26	Female	44	Yes	No	No
27	Male	43	Yes	No	No
28	Male	45	Yes	No	No
29	Female	44	Yes	No	No
30	Female	45	Yes	No	No
31	Male	40	Yes	No	No
32	Male	40	Yes	No	No
33	Female	41	Yes	No	No
34	Male	44	Yes	No	No
35	Male	45	Yes	No	No
36	Female	42	Yes	No	No
37	Female	45	Yes	No	No
38	Female	43	Yes	No	No
39	Male	40	Yes	No	No
40	Male	44	Yes	No	No
41	Male	45	Yes	No	No
42	Male	41	Yes	No	No
43	Male	42	No	No	No
44	Male	42	No	No	No
45	Female	40	No	No	No
46	Male	43	No	No	No
47	Male	42	No	No	No
48	Female	45	No	No	No
49	Female	42	No	No	No

50	Female	40	No	No	No
51	Female	40	No	No	No
52	Female	43	No	No	No

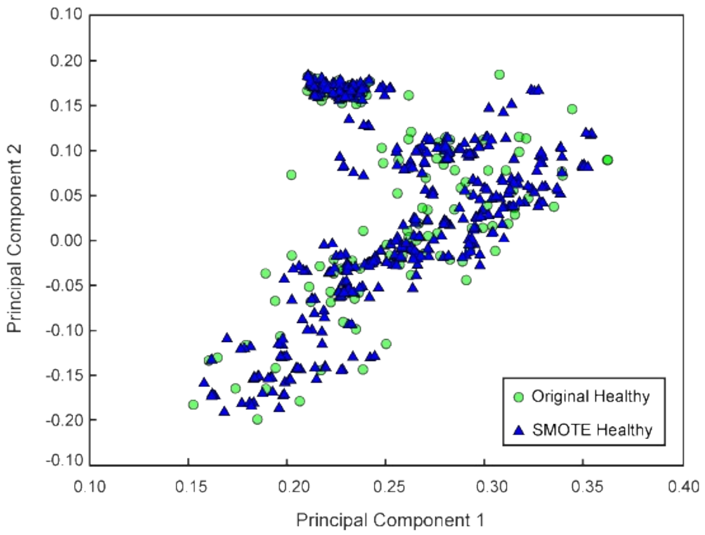

Figure S2. PCA analysis of original and synthetic data points from the healthy group.

**Table S2. 95% confidence intervals (CI) of model performance without and with SMOTE.**		
Model	95% CI without SMOTE	95% CI with SMOTE
KNN	0.89-0.94	0.94-0.97
ANN	0.78-0.85	0.92-0.96
QDA	0.81-0.90	0.90-0.96
SVM	0.50-0.59	0.89-0.95

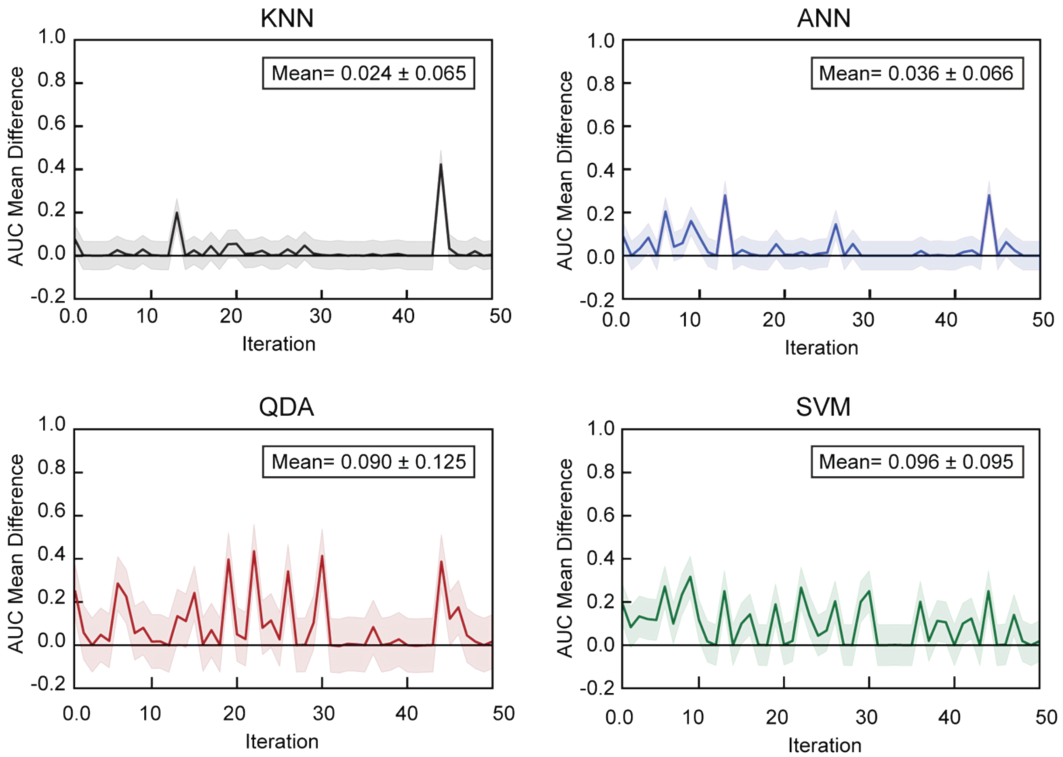

Figure S3. AUC mean difference plots of training and testing datasets without SMOTE-generated data for all models.

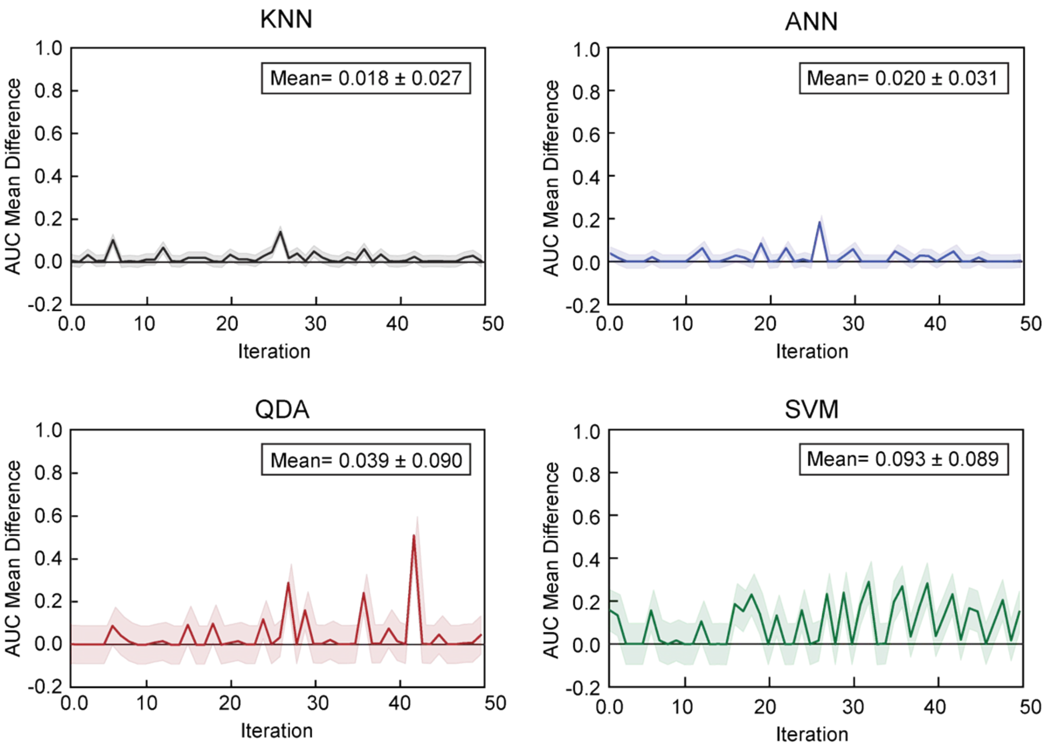

Figure S4. AUC mean difference plots of training and testing datasets with SMOTE-generated data for all models.
